# Microbial keratitis in Sao Paulo, Brazil: a 10-year review of laboratory results, epidemiological features, and risk factors

**DOI:** 10.5935/0004-2749.2022-0060

**Published:** 2023-09-27

**Authors:** Camila Kase, Yasmin Tournier Boppré, Talita Trevizani Rocchetti, Maria Cecília Zorat Yu, Arthur Gustavo Fernandes, Ana Luisa Hofling-Lima

**Affiliations:** 1 Department of Ophthalmology, Escola Paulista de Medicina, Universidade Federal de São Paulo, São Paulo, SP, Brazil

**Keywords:** Corneal ulcer/epidemiology, Cornea ulcer/microbiology, Keratitis, Eye infections, Úlcera da córnea/epidemiologia, Úlcera da córnea/microbiologia, Ceratite, Infecções oculares

## Abstract

**Purpose:**

To study epidemiological data, laboratory results, and risk factors associated with microbial keratitis.

**Methods:**

We conducted a retrospective study of corneal sample cultures from patients with microbial keratitis from January 2010 to December 2019. Results were analyzed according to the etiological diagnosis of bacterial, mycotic, or parasitic infection and were associated with related risk factors.

**Results:**

We analyzed 4810 corneal samples from 4047 patients (mean age 47.79 ± 20.68 years; male 53.27%). The prevalence of bacterial, fungal, and *Acanthamoeba* infections were 69.80%, 7.31%, and 3.51%, respectively. The most frequently isolated bacteria were coagulase-negative *Staphylococcus* (CoNS) (45.14%), *S. aureus* (10.02%), *Pseudomonas* spp. (8.80%), and *Corynebacterium* spp. (6.21%). Among CoNS, the main agent was *S. epidermidis* (n=665). For mycotic keratitis, *Fusarium* spp. (35.42%) and *Candida parapsilosis* (16.07%) were the most common agents among filamentous and yeasts isolates, respectively. Contact lens use was associated with a positive culture for *Acanthamoeba* spp. (OR = 19.04; p < 0.001) and *Pseudomonas* spp. (OR = 3.20; p < 0.001). Previous ocular trauma was associated with positive fungal cultures (OR = 1.80; p = 0.007), while older age was associated with positive bacterial culture (OR = 1.76; p = 0.001).

**Conclusions:**

Our findings demonstrated a higher positivity of corneal sample cultures for bacteria. Among those, CoNS was the most frequently identified, with *S. epidermidis* as the main agent. In fungal keratitis, *Fusarium* spp. was the most commonly isolated. Contact lens wearers had higher risks of positive cultures for *Acanthamoeba spp.* and *Pseudomonas* spp. Ocular trauma increased the risk of fungal infection, while older age increased the risk of bacterial infection.

## Abstract (pt)

**Objetivo:**

Estudar os dados epidemiológicos, resultados laboratoriais e fatores de risco associados às ceratites infecciosas.

**Métodos:**

Estudo retrospectivo das amostras de cultura de córnea em pacientes com ceratites infecciosas entre Janeiro/2010 a Dezembro/2019. Os resultados foram analisados de acordo com o diagnóstico etiológico de infecção bacteriana, fúngica ou parasitária e correlacionado com os fatores de risco relacionados.

**Resultados:**

Quatro mil, oitocentas e dez amostras corneanas de 4047 pacientes (média de idade de 47,79 ± 20,68 anos; homens em sua maioria (53,7%) foram incluídas. A prevalência de infecções por bactéria, fungo e *Acanthamoeba* foram de 69.80%, 7,31%, and 3,51%, respectivamente. A maioria das bactérias mais frequentemente isoladas foram Staphylococcus coagulase-negativo (CoNS) (45,14%), *S. aureus* (10,02%), *Pseudomonas* spp. (8,80%), e *Corynebacterium* spp. (6,21%). Dentre CoNS, o principal agente foi *S. epidermidis* (n = 665). Nas ceratites fúngicas, *Fusarium* spp. (35,42%) e *Candida parapsilosis* (16,07%) foram os agentes mais comuns entre os filamentosos e leveduriformes, respectivamente. O uso de lentes de contato foi associado à cultura positiva para *Acanthamoeba* spp. (OR = 19,04; p < 0,001) e *Pseudomonas* spp (OR = 3,20; p < 0,001). Trauma ocular prévio foi associado a culturas positivas para fungo (OR = 1,80; p = 0,007), e idade avançada foi associada a culturas positivas para bactéria (OR = 1,76; p = 0,001).

**Conclusões:**

Nossos achados demonstraram uma maior positividade para bactérias em amostras de cultura corneana. Dentre estas, CoNS foi mais frequentemente identificado, sendo *S. epidermidis* o principal agente. Nas ceratites fúngicas, *Fusarium* spp. Foi o mais comumente isolado. O risco de positividade para *Acanthamoeba spp.* e *Pseudomonas* spp. foi maior em usuários de lentes de contato. Trauma ocular aumentou o risco de cultura positiva para fungo, ao passo que idade mais avançada aumentou o risco de infecção bacteriana.

## INTRODUCTION

Infectious keratitis is a severe corneal disease that can lead to visual impairment due to ocular complications such as corneal scars, endophthalmitis, and globe perforation^(1^, ^2)^. Early clinical evaluation and treatment are critical to achieving the best visual prognosis^(3)^.

Infectious microbial keratitis is a corneal disease with distinct etiologies whose primary causative agents are bacteria, fungi, and protozoa^(3)^. Bacteria are the most common agents in the developed world, but fungal etiologies are also important causative agents^(2,4)^.

In the United States, 930,000 outpatient consultations are performed annually for infectious keratitis, with 58,000 emergency department visits^(5)^. The incidence of keratitis varies according to the studied population; it is higher in the developing world than in the developed world^(2)^.

Determining the etiological agent based on clinical signs is challenging, even for corneal specialists^(6)^. However, the presence of risk factors may guide the diagnostic process. Contact lenses are an important risk factor for infectious keratitis; in the developing world, eye trauma from organic material encountered during work in rural areas is commonly associated with keratitis^(7,8)^. Other reported risk factors include previous ocular surgery, ocular surface disease, herpetic keratitis, systemic di sease, and topical corticosteroids^(9,10)^.

Probable etiological agents responsible for infectious keratitis vary depending on the geographic location. Estopinal et al. reviewed bacterial and fungal agents in various regions of the US and found a more significant proportion of bacterial and fungal keratitis in northern and southern locations, respectively^(11)^. Yeasts were more prevalent in colder regions, and molds were predominant in warmer places.

Brazilian cities differ in socioeconomics, culture, and environment; therefore, the predominance of etiological agents should vary depending on the study location. Moreover, the complexity of the centers where the studies are conducted should be considered because university hospitals usually have more severe cases than secondary hospitals. In a study of Sao Paulo City, there was a higher prevalence of bacterial keratitis^(10)^; by contrast, fungi were the primary agents in Uberlandia, a city where patients involved in agricultural activity are referred^(12)^.

Infectious keratitis can affect patients of all ages^(13,14)^. In studies conducted at the Ophthalmology Department of *Escola Paulista de Medicina/Hospital São Paulo*, the mean age was 42.4 years, with a higher prevalence in males^(10)^.

Diagnosing microbial keratitis through clinical evaluation is necessary but inconclusive. Diagnosis requires laboratory evaluations such as corneal scraping or biopsy^(15)^, especially when there are signs of severe keratitis^(16)^. Examination modalities such as in vivo confocal microscopy and corneal optical coherence tomography may help in atypical or complex cases where the corneal culture is inconclusive^(17)^. In vivo confocal microscopy is essential for detecting infectious keratitis caused by fungus and *Acanthamoeba* spp.^(18)^.

The present study is a retrospective review of the epidemiological data and laboratory results of corneal culture scrapes of patients clinically suspected to have infectious keratitis.

## METHODS

The laboratory results of corneal scrape samples taken from patients with infectious keratitis, from January 2010 to December 2019, were analyzed retrospectively using information contained in the database of the Microbiology Laboratory of the Department of Ophthalmology of *Escola Paulista de Medicina, Universidade Federal de São Paulo*, Brazil. We also reviewed the medical records. The Institutional Ethics Committee Review Board of *Universidade Federal de São Paulo* approved the study. The research was conducted in accordance with the Declaration of Helsinki.

Every available corneal scrape sample from January 2010 to December 2019 was included in this study, excluding corneal scrapes with no available results. The corneal sample harvest methodology was used according to Cariello et al.^(10)^.

Laboratory results were analyzed according to the etiological diagnosis. They were classified as bacterial, mycotic, and parasitic. They were correlated with epidemiological data, including sex, age, the affected eye, duration of symptoms, and related risk factors (contact lens wear, previous ocular surgery, and previous ocular trauma). The presence of risk factors was evaluated retrospectively from the database of the Microbiology Laboratory of the Department of Ophthalmology of *Escola Paulista de Medicina, Universidade Federal de São Paulo*, Brazil, and the electronic medical records.

This retrospective study aimed to analyze the epidemiological data of patients with microbial keratitis and the laboratory results of their corneal scrapes and to identify possible risk factors for corneal infection.

Statistical analysis was performed using STATA 14.0 (StataCorp LP, College Station, TX, USA). Frequency tables were used for descriptive analysis. Multiple logistic regressions were used to calculate the effect of covariates on the evaluated outcomes. For all tests, a significant p-value was considered less than or equal to 0.05.

## RESULTS

From January 2010 to December 2019, 4810 corneal samples were collected from 4047 patients. The number of samples per patient varied from 1 to 14, with a mean of 1.19 ± 0.58 and a median of 1. Table 1 shows the number of samples for each patient. The mean interval between two samples from the same eye was 22 ± 34 (range, 1-364) days, with a median of 11 days (Table 2). Long intervals, such as 364 days, occurred in patients with *Acanthamoeba* infection, with recurrent positivity even after treatment. Individuals with more than one sample were those who underwent reculture when the previous cultures were negative. There were 41 cases of reculture when there was no improvement of the corneal lesion, despite specific treatment based on culture results. In 31/41 cases (75.61%), the agent isolated in the second culture was different from that in the first culture; in 2/41 cases (4.88%), it was the same agent, and in 8/41 cases (19.51%), the second culture was negative. The studied population had 992 (24.51%) contact lens wearers.

**Table 1:** Number of corneal samples for each patient

Number of corneal samples	n (%)
1	3507 (86.66)
2	392 (9.69)
3	101 (2.50)
4	34 (0.84)
≥5	13 (0.31)
TOTAL	4047 (100.00)

**Table 2:** Interval of two samples in cases of reculture (n=638)

Descriptive, days	
Mean	22
Median	11
Standard deviation	34
Minimum	1
Maximum	364
Distribution, cases	
0-7 days	235
7-30 days	284
30-60 days	74
60-90 days	17
>90 days	28

The patients had a mean age of 47.79 ± 20.68 years (range, 15 days to 102 years) and a median of 47 years, with 2156 male patients (53.27%) and 1891 female patients (46.73%). The right and left eyes were affected in 2017 (49.84%) and 2030 (50.16%) patients, respectively. Table 3 displays the general characteristics. Symptom duration until ophthalmological care ranged from 1 to 2190 days, with a mean of 22.01 ± 84.50 days and a median of 7 days.

**Table 3:** General characteristics of the patients

	n (%)
Sex	
Female	1891 (46.73)
Male	2156 (53.27)
Age (years)	
00-18	230 (5.68)
19-59	2576 (63.65)
60+	1241 (30.66)
Use of topical antibiotics*	
Yes	1544 (38.40)
No	2492 (61.60)
Previous ocular surgery*	
Yes	999 (24.68)
No	3048 (75.32)
Contact lens wear*	
Yes	992 (24.68)
No	3048 (75.32)
Eye trauma*	
Yes	261 (6.45)
No	3786 (93.55)
Eye trauma with organic material*	
Yes	62 (1.53)
No	3985 (98.47)
Keratoprosthesis*	
Yes	19 (0.47)
No	4028 (99.53)
Duration of symptoms	
≤1 week	1434 (35.43)
>1 week	2613 (64.57)
Total	**3682 (100.00)**

* Characteristics of the scraped eye.

The prevalence of a positive bacteria culture was 69.80% (95% confidence interval [CI]: 68.37-71.20%); for fungi, it was 7.31% (95% CI: 6.55-8.16%), and for *Acanthamoeba,* it was 3.51% (95% CI: 2.98-4.12%) (Table 4).

**Table 4:** Prevalence of positive microbial cultures in the population

Microorganism	n	Prevalence (95% confidence interval)
Bacteria	2825	69.80% (68.37-71.20%)
Fungi	296	7.31% (6.55-8.16%)
Acanthamoeba	142	3.51% (2.98-4.12%)
Any positive culture	3050	75.36% (74.01-76.67%)

Of the 4810 samples, 3376 showed positive cultures for at least one microorganism, representing an overall culture positivity frequency of 70.2%. The frequency of positive cultures was greatest for bacteria (n=3062/4810, 63.66%), followed by fungi (n=334/4810, 6.94%) and *Acanthamoeba* (n=170/4810, 3.53%). A total of 118 cultures (2.45%) were simultaneously positive for bacteria and fungi, 69 (1.43%) were simultaneously positive for bacteria and *Acanthamoeba*, and four (0.08%) were simultaneously positive for fungi and *Acanthamoeba*. One culture (0.02%) was simultaneously positive for bacteria, fungi, and *Acanthamoeba*.

Among the 4810 corneal samples, 4639 (96.44%) were sent for bacterial investigation. Among the total samples tested, 3062/4639 (66.01%) showed positive bacterial cultures, with a mean of 1.17 ± 0.43 bacteria (2594 cultures with one bacterium, 408 with two bacteria, 59 with three bacteria, and one with four bacteria). A total of 3591 bacteria were detected. Among the bacteria, 2741 were gram-positive (76.33%), and 835 were gram-negative (23.25%). Ten were mycobacteria (0.28%), and five could not be identified (0.14%). The most frequently isolated bacteria were coagulasenegative *Staphylococcus* (CoNS) (n = 1621, 45.14%), *S. aureus* (n=360, 10.02%), *Pseudomonas* spp. (n=316, 8.80%) and *Corynebacterium* spp. (n=223, 6.21%). Among CoNS, the main isolated species was *S. epidermidis* (n=665). The distribution of the identified bacteria is displayed in Table 5. Multivariate regression analysis revealed an association between a positive bacterial culture and sex, age, topical antibiotic use, and complaint time.

**Table 5:** Classification of identified bacterial cultures

Microorganism	Positive cultures n (%)
**Gram-positive cocci**	
*Staphylococcus*	
*Coagulase-negative Staphylococcus*	1621 (45.14)
*Staphylococcus epidermidis*	665 (18.52)
*Staphylococcus hominis*	107 (2.98)
*Staphylococcus haemolyticus*	69 (1.92)
*Staphylococcus warneri*	71 (1.98)
*Staphylococcus saprophyticus*	23 (0.64)
*Staphylococcus lugdunensis*	13 (0.36)
*Other CoNS*	145 (4.04)
*Unidentified CoNS*	528 (14.70)
*Staphylococcus aureus*	360 (10.02)
Other *Staphylococcus*	5 (0.14)
*Staphylococcus spp.*	1 (0.03)
**Total Staphylococcus**	**1987 (55.33)**
*Streptococcus*	
*Streptococcus pneumoniae*	177 (4.93)
*Streptococcus viridans*	109 (3.03)
*Streptococcus pyogenes*	8 (0.22)
*Streptococcus spp.*	15 (0.42)
**Total Streptococcus**	**309 (8.60)**
*Micrococcus*	
*Micrococcus luteus*	47 (1.31)
*Micrococcus lylae*	6 (0.17)
*Micrococcus spp.*	17 (0.47)
**Total Micrococcus**	**70 (1.95)**
*Enterococcus*	
*Enterococcus faecalis*	17 (0.47)
*Enterococcus faecium*	2 (0.05)
*Enterococcus spp.*	2 (0.05)
**Total Enterococcus**	**21 (0.58)**
**Gram-negative bacilli**	
*Pseudomonas*	
*Pseudomonas aeruginosa*	285 (7.93)
*Pseudomonas spp.*	31 (0.86)
**Total Pseudomonas**	316 (8.80)
*Serratia spp.*	171 (4.76)
*Acinetobacter spp.*	27 (0.75)
*Enterobacter spp.*	29 (0.81)
*Proteus spp.*	26 (0.72)
*Klebsiella spp.*	19 (0.53)
*Morganella morganii*	18 (0.50)
*Citrobacter spp.*	16 (0.44)
*Stenotrophomonas maltophilia*	10 (0.28)
Unidentified	2 (0.06)
**Gram-positive bacilli**	
*Corynebacterium spp.*	223 (6.21)
*Bacillus spp.*	38 (1.06)
*Propionibacterium spp.*	19 (0.53)
Others	9 (0.25)
Unidentified	29 (0.81)
**Others**	
*Moraxella spp.*	117 (3.26)
*Haemophilus spp.*	22 (0.61)
*Mycobacterium spp.*	10 (0.28)
*Neisseria spp.*	5 (0.14)
*Nocardia spp.*	2 (0.05)
Others	96 (2.67)
Total	3591 (100.00)

CoNS: Coagulase-negative *Staphylococcus.*

Males were 1.22 times more likely to have a positive bacterial culture than females (odds ratio [OR] = 1.22; 95% CI: 1.06-1.41; p=0.006), and individuals aged ≥60 years were 1.76 times more likely to have a positive bacterial culture than individuals aged <18 years (OR=1.76; 95% CI: 1.29-2.40; p=0.001. By contrast, individuals who used topical antibiotics were 0.48 times more likely to have a positive bacterial culture than individuals who did not (OR=0.48; 95% CI: 0.39-0.58; p<0.001); and individuals with complaints of ≤ 1 week had 0.72 times the chance of having a positive bacterial culture than individuals with complaints of > 1 week (OR=0.72; 95% CI: 0.62-0.84; p<0.001).

*Pseudomonas* spp. was the third most frequent bacterial subtype found in positive cultures (8.80%). The regression analysis revealed an association between positive culture for *Pseudomonas* spp. and contact lens use, topical antibiotics use, and complaint time. Individuals who wore contact lenses were 3.20 times more likely to have a positive culture for *Pseudomonas* spp. than individuals who did not (OR=3.20; 95% CI: 2.44-4.19; p<0.001). By contrast, individuals who used topical antibiotics were 0.43 times more likely to have a positive culture for *Pseudomonas* spp. than individuals who did not (OR=0.43; 95% CI: 0.30-0.62; p<0.001). Individuals with complaints of ≤1 week had 0.46 times the chance of having a positive culture for *Pseudomonas* than individuals with complaints of >1 week (OR=0.46; 95%CI: 0.36-0.59; p<0.001).

Of the 4810 corneal samples, 4426 (92.02%) were sent for fungal investigation. Among the total tested, 334/4426 (7.55%) had positive fungal cultures, with 227 and 109 cultures of filamentous fungi and yeasts, respectively. There were two cases of filamentous fungi and yeast coinfection for a total of 336 identified microorganisms. The distribution of the identified fungi is presented in table 6.

**Table 6:** Classification of identified fungal cultures

Microorganism	Positive cultures, n (%)
**Filamentous**	**227 (67.56)**
*Fusarium*	
*Fusarium solani species complex*	99 (29.46)
*Fusarium dimerum species complex*	8 (2.38)
*Fusarium oxysporum species complex*	7 (2.08)
*Fusarium spp.*	5 (1.49)
Total Fusarium	119 (35.42)
*Aspergillus*	
*Aspergillus flavus species complex*	15 (4.46)
*Aspergillus fumigatus species complex*	12 (3.57)
*Aspergillus spp.*	3 (0.89)
Total Aspergillus	30 (8.93)
*Penicillium spp.*	12 (3.57)
*Curvularia spp.*	10 (2.98)
*Paecilomyces spp.*	11 (3.27)
*Acremonium spp.*	5 (1.49)
*Colletotrichum spp.*	5 (1.49)
*Chaetomium spp.*	3 (0.89)
*Mycelia sterilia*	3 (0.89)
*Scedosporium spp.*	6 (1.79)
*Purpureocillium lilacinus*	4 (1.19)
Others	16 (4.76)
Not identified	5 (1.49)
**Yeasts**	**109 (32.44)**
*Candida parapsilosis*	54 (16.07)
*Candida albicans*	25 (7.44)
*Candida guilliermondii*	12 (3.57)
Others	18 (5.36)
**Total**	**336 (100.00)**

Multivariate regression analysis revealed associations between a positive fungal culture and topical antibiotics use, previous ocular surgery, ocular trauma, contact lens use, and complaint time. Individuals who used topical antibiotics were 2.10 times more likely to have a positive fungal culture than individuals who did not (OR=2.10; 95% CI: 1.62-2.76; p<0.001). Individuals with previous ocular surgery were 1.46 times more likely to have a positive fungal culture than individuals without (OR=1.46; 95% CI: 1.10-1.93; p=0.008). Individuals with ocular trauma were 1.80 times more likely to have a positive fungal culture than individuals without (OR=1.80; 95% CI: 1.17-2.77; p=0.007). Individuals with complaints of ≤1 week were 1.69 times more likely to have a positive fungal culture than those with complaints of > 1 week (OR=1.69; 95% CI: 1.25-2.29; p=0.001). Conversely, individuals who wore contact lenses were 0.67 times more likely to have a positive fungal culture than individuals who did not (OR=0.67; 95% CI: 0.47-0.94; p=0.020).

Of the 4810 corneal samples, 1784 (37.09%) were sent for investigation for *Acanthamoeba*. Among the total tested, 170/1784 (9.53%) had positive cultures. Multivariate regression analysis revealed associations between positive culture for *Acanthamoeba* and contact lens use and complaint time. Individuals who wore contact lenses had 19.04 times the chance of having a positive culture for *Acanthamoeba* than individuals who did not (OR=19.04; 95% CI: 10.08-35.97; p<0.001). Individuals with complaints of ≤1 week had 10.82 times the chance of having a positive culture for *Acanthamoeba* than individuals with complaints of >1 week (OR=10.82; 95% CI: 5.37-21.81; p<0.001).

## DISCUSSION

The overall positive culture rate of this study was 70.2%, which is similar to values found in other countries (range, 34.2%-78.3%)^(19,20)^ and in different regions of Brazil (range, 26.3% to 87.2%)^(21,22)^ (Tables 7 and 8). The variability among countries may be due to the most prevalent etiological agents in each country, sampling methodology, and different standardizations of the media used for corneal culture.

**Table 7:** Distribution of the main isolated bacteria, total number of corneal scrapes sent for culture, and overall culture positivity of studies conducted in other countries

Setting	Duration	*S. aureus* n (%)*	*Corynebacterium spp.* n (%)*	*Pseudomonas spp.* n (%)*	*S. epidermidis* n (%)*	Coagulase-negative *Staphylococcus* n (%)*	*Serratia spp.* n (%)*	*S. pneumoniae* n (%)*	Gram-positive n (%)*	Gram-negative n (%)*	Total corneal scrapes sent to culture	Culture positivity (%)	Article DOI
**Portugal, University of Porto**	Sep 2007 - Aug 2015 (8 years)	**15 (23.1)**	13 (20.0)	9 (13.8)	3 (4.6)	6 (9.2)	6 (9.2)	7 (10.8)	**46 (70.8)**	19 (29.2)	235	38.4	10.1097/ICL.0000000000000298
**Mexico, Instituto de Oftalmologia “Conde de Valenciana”**	Jan 2002 - Dec 2011 (10 years)	53 (9)	38 (6)	79 (13)	**151 (25)**	63 (10)	13 (2)	15 (2)	**412 (67.0)**	132 (21.0)	1638	38.0	10.1097/ICO.0000000000000428
**India, Joseph Eye Hospital**	Jan 2005 - Dec 2012 (8 years)	235 (19.5)	4 (0.3)	119 (9.7)	534 (44)	**542 (45)**	1 (0.08)	140 (11.6)	**992 (82.3)**	213 (17.7)	2170	77.0	10.1155/2013/181564
**UK, East Kent Hospitals University National Health Service Foundation Trust**	Jan 1999 - Dec 2008 (10 years)	24 (14.8)	NI	**80 (49.4)**	NI	13 (8)	5 (3.1)	9 (5.6)	63 (38.9)	**99 (61.1)**	476	34.2	10.1016/j.ophtha.2011.04.021
**South Korea, Seoul National University**	Jan 2007-Dec 2016 (10 years)	13 (12.1)	5 (5.6)	13 (12.1)	9 (8.4)	**17 (15.9)**	4 (3.7)	9 (8.4)	**69 (64.5)**	38 (35.5)	129	78.29	10.1371/journal.pone.0213103
**Japan, Juntendo University School of Medicine**	Jan 1999-Dec 2003 (5 years)	14 (13.7)	13 (12.7)	1 (1)	29 (28.4)	**29 (33.3)**	16 (15.7)	4 (3.9)	**77 (75.5)**	18 (17.6)	123	58.5	10.1097/01.icl.0000237825.98225.ca
**Spain, University Hospital of Guadalajara**	Jan 2010-Dec 2016 (7 years)	24 (9.40)	25 (9.8)	14 (5.5)	NI	**73 (28.6)**	0	9 (3.5)	**210 (82.4)**	31 (12.2)	297	64.4	10.7883/yoken.JJID.2018.269
**USA, Saint Louis University School of Medicine**	1999-2013 (15 years)	40 (14.0)	NI	**60 (21.0)**	NI	47 (16.0)	9 (3.0)	NI	**137 (48.0)**	96 (34.0)	416	74.0	10.1016/j.ajo.2018.09.032
**Taiwan, Chang Gung Memorial Hospital**	Jan 2003-Dec 2012 (10 years)	87 (8.4)	NI	**253 (24.4)**	NI	166 (16.6)	54 (5.2)	30 (2.9)	**533 (41.6)**	506 (39.5)	2012	49.3	10.1097/ICO.0000000000000734
**USA, UT Southwestern Medical Center**	Sep 2009-Aug 2014 (5 years)	18 (8.2)	4 (1.8)	35 (15.9)	9 (4.1)	**36 (16.4)**	6 (2.7)	13 (5.9)	**102 (46.4)**	85 (38.6)	232	66.0	10.4172/2155-9570.1000498
**Toronto, University Health Network, University of Toronto**	Jan 2000-Dec 2010 (11 years)	154 (17)	NI	91 (10)	NI	**328 (37)**	28 (3)	NI	**684 (76.2)**	213 (23.8)	1701	57.4	10.1016/j.ophtha.2012.03.031
**New Zealand, Waikato Hospital**	Jan 2003-Dec 2007 (5 years)	20 (11.5)	12 (6.9)	7 (4)	NI	**71 (40.8)**	2 (1.1)	13 (7.5)	**136 (78.2)**	35 (20.2)	265	65.6	10.1111/j.1442-9071.2010.02480.x

* Percentage of this bacteria species out of the total microorganisms.

NI= no information.

**Table 8:** Distribution of the main isolated bacteria, total number of corneal scrapes sent for culture, and overall culture positivity of studies conducted in Brazilian ophthalmological services

Setting	Duration	*S. aureus* n (%)*	Corynebacterium *spp.* n (%)*	*Pseudomonas spp.* n (%)*	*S. epidermidis* n (%)*	Coagulase-negative *Staphylococcus* n (%)*	*Serratia spp.* n (%)*	*S. pneumoniae* n (%)*	Gram-positive n (%)	Gram-negative n (%)	Total corneal scrapes sent for culture	Culture positivity (%)	Article DOI
**São Paulo, São Paulo, Hospital São Paulo**	Jul 1975 - Sep 2007 (32 years)	661 (20.0)	225 (6.8)	369 (11.1)	34 (1.0)	**824 (24.9)**	86 (2.6)	234 (7.1)	**1231 (55.6)**	587 (26.5)	6804	48.6	10.1007/s10792-011-9441-0
**Sorocaba, São Paulo, Hospital Oftalmológico de Sorocaba**	2005 - 2009 (5 years)	**81 (29.1)**	0	25 (9.0)	**81 (29.1)**	NI	1 (0.36)	NI	**191 (72.08)**	74 (27.92)	963	28.86	10.5935/0034-7280.20170024
**Ribeirão Preto, São Paulo, Ophthalmologic Clinic of São Paulo University**	Oct 2003 - Sep 2006 (3 years)	10 (13.7)	0	4 (5.5)	**13 (17.8)**	NI	4 (5.5)	8 (11.0)	**42 (75.00)**	14 (25.00)	118	61.00	10.34117/bjdv6n4-226
**Uberlândia, Minas Gerais, Clinical Hospital of Federal University of Minas Gerais**	Jul 2001 - Aug 2004 (3 years)	2 (6.2)	0	3 (9.4)	0	0	0	**7 (21.9)**	**9 (64.29)**	5 (35.71)	65	49.23	10.1590/s0004-27492011000100007
**Curitiba, Paraná, Hospital Universitário Evangélico de Curitiba and Hospital de Olhos do Paraná**	Jun 2014 - Apr 2016 (2 years)	1 (5.0)	0	**3 (15.0)**	1 (5.0)	NI	1 (5.0)	2 (10.0)	4 (44.5)	**5 (55.5)**	63	31.75	10.1590/s0004-27492011000100002
**São Luís, Maranhão, São Francisco Eye Center**	Jun 2007 - Jun 2015 (8 years)	20 (12.3)	2 (1.2)	**29 (17.8)**	1 (0.6)	5 (3.1)	0	0	30 (35.71)	**54 (64.29)**	187	87.17	10.4274/tjo.galenos.2021.98046
**Natal, Rio Grande do Norte, Hospital Universitário Onofre Lopes**	Jan 2016 - Dez 2017 (2 years)	5 (17.2)	0	**13 (44.8)**	NI	4 (13.8)	0	1 (3.4)	10 (34.48)	**19 (65.52)**	190	26.32	10.1177/112067211002000312

* Percentage of this bacteria species out of the total microorganisms.

NI= no information.

We demonstrated that bacteria were the primary agents responsible for infectious keratitis diagnosed by laboratory examinations, followed by fungi and *Acanthamoeba*. The most frequently isolated bacteria were CoN*S* (45.14%)*, S. aureus* (10.02%), *Pseudomonas* spp. (8.80%), and *Corynebacterium* spp. (6.21%). Other studies also showed CoN*S* as the primary bacterial agent (Table 7). *Pseudomonas* spp. was the most common cause of infectious bacterial keratitis in studies conducted in the United Kingdom, Saint Louis in the US, and Taiwan^(19,23,24)^. The predominance of bacteria as the main etiological agent of microbial keratitis has also been noted in other countries (Table 8).

Brazil is a large country with socioeconomic, cultural, and climatic diversity. The comparison of the etiological agents of infectious keratitis between our study, located in Sao Paulo, and studies conducted in other regions of Brazil shows a predominance of bacterial keratitis in the country (Table 9), except in Uberlandia, where there is a higher prevalence of fungal etiology^(10^, ^12)^. This may be because Uberlandia has a medical service that is a referral center for patients that work in agriculture, who are possibly more susceptible to eye trauma from organic material. Gram-positive organisms were the primary causative agents of infectious keratitis in the Southeast, and gram-negative organisms were most common in the Northeast and South of Brazil (Table 8). Walkden et al. demonstrated that summer and warmer temperatures are favorable for the growth of *Pseudomonas* spp.^(25)^. This could be the reason for the predominance of gramnegative organisms, mainly *Pseudomonas* spp., in the Northeast region, which has a warmer climate than the southern region of Brazil. There is only one hospital with study documentation in the south of the country (Curitiba, Paraná), where the weather is colder but due to the limited cases (only 9 positive cultures), it was hard to infer the difference in prevalence. The influence of the regional temperature needs to be evaluated by studies with a larger sample size.

**Table 9:** Frequency of etiological agents (bacteria, fungi, and *Acanthamoeba*) in ophthalmological services in Brazil and other countries

Setting	Bacteria n (%)	Fungi n (%)	*Acanthamoeba* n (%)	Total N	Article DOI
**Brazil**					
São Paulo, São Paulo, Hospital São Paulo	**2699 (39.70)**	364 (5.30)	246 (3.60)	3309	10.1007/s10792-011-9441-0.
Sorocaba, São Paulo, Hospital Oftalmológico de Sorocaba	**265 (95.33)**	13 (4.67)	0	278	10.5935/0034-7280.20170024
Ribeirão Preto, São Paulo, Ophthalmologic Clinic of São Paulo University	**56 (76.71)**	17 (23.39)	0	73	10.34117/bjdv6n4-226
Uberlândia, Minas Gerais, Clinical Hospital of Federal University of Minas Gerais	14 (43.75)	**18 (56.25)**	0	32	10.1590/s0004-27492011000100007
Curitiba, Paraná, Hospital Universitário Evangélico de Curitiba and Hospital de Olhos do Paraná	**9 (45.00)**	8 (40.00)	3 (15.00)	20	10.1590/s0004-27492011000100002
São Luís, Maranhão, São Francisco Eye Center	**84 (44.91)**	56 (30.00)	2 (1.60)	163	10.4274/tjo.galenos.2021.98046
Natal, Rio Grande do Norte, Hospital Universitário Onofre Lopes	**29 (58.00)**	21 (42.00)	0	50	0.1177/112067211002000312
**Other countries**					
Mexico, Instituto de Oftalmologia “Conde de Valenciana”	**544 (88.31)**	72 (11.68)	1 (0.16)	616	10.1097/ICO.0000000000000428
India, Joseph Eye Hospital	**807 (48.47)**	493 (29.61)	9 (0.54)	1665	10.1155/2013/181564
UK, East Kent Hospitals University National Health Service Foundation Trust	**162 (94.20)**	5 (2.90)	5 (2.90)	172	10.1016/j.ophtha.2011.04.021
Japan, Juntendo University School of Medicine	**95 (93.14)**	6 (5.88)	1 (1.05)	102	10.1097/01.icl.0000237825.98225.ca
USA, Saint Louis University School of Medicine	**233 (81.47)**	45 (16.00)	7 (2.45)	286	10.1016/j.ajo.2018.09.032
Taiwan, Chang Gung Memorial Hospital	**1039 (81.10)**	205 (16.00)	14 (1.10)	1282	10.1097/ICO.0000000000000734
USA, UT Southwestern Medical Center	**187 (85.00)**	32 (14.50)	1 (0.50)	220	10.4172/2155-9570.1000498
Toronto, University Health Network, University of Toronto	**897 (91.80)**	59 (6.00)	21 (2.20)	977	10.1016/j.ophtha.2012.03.031
New Zealand, Waikato Hospital	**171 (98.30)**	3 (1.70)	0	174	10.1111/j.1442-9071.2010.02480.x

The most frequently isolated fungi were *Fusarium spp.,* a filamentous agent, and *Candida parapsilosis* (a yeast). Studies in other countries also showed that *Fusarium* spp. were the most commonly isolated fungi; however, unlike our findings, *Aspergillus* spp. was the second most commonly isolated fungus, followed by *Candida spp.*^(4^, ^24)^. Cariello et al.^(10)^ found that, in our setting from 1975 to 2007, *C. albicans* was the primary agent in the yeast group of fungi. In contrast, we showed that *C. parapsilosis* was the most common. This change could be due to the increase of *C. parapsilosis* as a human pathogen in recent decades^(26)^.

The distribution of *C. parapsilosis* over the study period was 5 cases in 2010, 4 cases in 2011, 4 cases in 2012, 6 cases in 2013, 3 cases in 2014, 6 cases in 2015, 10 cases in 2016, 7 cases in 2017, 2 cases in 2018, and 7 cases in 2019. These patients were not hospitalized. Sample contamination by the hands of healthcare workers may have occurred during the corneal scraping, although the entire procedure is performed with caution (the air conditioning and fans are turned off, with proper handwashing and use of procedure gloves). Nevertheless, we found 6 cases (6/53, 11.32%) of *C. parapsilosis* that were treated without specific therapy for fungal infection during our study period.

The association between the complaint time until ophthalmological care and the etiological agent of infectious keratitis was not as stated in previous studies. We found that individuals with ocular symptoms lasting ≤1 week had an increased risk of keratitis caused by fungi and *Acanthamoeba* and a decreased risk of bacterial keratitis. Mascarenhas et al. found that patients with *Acanthamoeba* were more likely to have a longer duration of symptoms than patients with bacterial or fungal keratitis^(27)^. In our study, atypical fungal infections with a ≤1-week history occurred in severe post-trauma, foreign body, and post-surgical cases. Studies with larger sample sizes would help elucidate these study differences.

In the present study, individuals who wore contact lenses were 19.04 and 3.20 times more likely to have positive cultures for *Acanthamoeba* and *Pseudomonas*, respectively, than individuals who did not. Contact lenses have been associated with positivity for *Acanthamoeba*^(28)^ and *Pseudomonas*^(29)^. The number of persons who wore contact lens for 10 years was 992 (24.51%). A previous study in our service the focused on a period of >32 years noted 868 contact lens wearers (12.8%)^(10)^, suggesting an increase in the number of contact lens wearers or that a more significant number of patients diagnosed with infectious keratitis wore contact lenses.

An association was found between positive culture for fungi and ocular trauma. This association could be explained by the fact that many Brazilians work in civil construction and are at risk of eye trauma accidents. Previous ocular surgeries, such as penetrating kerato-plasty and refractive surgery, were also associated with a positive culture for fungi, as shown in other studies^(3)^. Corticosteroids predispose to fungal keratitis, and their use is associated, in cases of proven fungal keratitis, with deeper infiltrates, worse disease progression, and worse treatment outcomes. In a previous study, cases of ocular trauma that developed *Fusarium* keratitis after the unmonitored use of topical antibiotic-corticosteroids were highlighted, demonstrating the possible relationship between corticosteroid treatment and mycotic keratitis in traumatized eyes. Ocular surgeries, such as penetrating keratoplasty, change the anatomy, increa se susceptibility to infections, and require long-term corticosteroid use, which could predispose to keratitis caused by these microorganisms^(30)^.

Corneal sample cultures with antibiotic sensitivity studies are used to diagnose etiological agents and guide treatment, especially when the patient does not respond satisfactorily to initial empirical treatment^(16)^. Furthermore, investigating the most prevalent microorganisms causing infectious keratitis can help ophthalmologists who do not have access to a microbiology laboratory or high-cost examinations such as confocal microscopy or optical coherence tomography to start treatment based on the most probable microbial agents.

The principal limitations of our study were related to the possibility of false-positive and false-negative laboratory results and the retrospective design of the study. False-positive results may be due to non-pathogenic bacteria from the patient’s microbiota, and false-negative results may be caused by insufficient material obtained by corneal scrape. Another limitation is the sample selection bias due to the higher prevalence of severe cases in university tertiary centers.

Microbial keratitis is a significant cause of blindness worldwide^(2)^. Treatment costs are often high, and patients may not have access to adequate follow-up either because of the cost of medications or transportation costs to an ophthalmological consultation^(9)^. In addition, this study found that patients required a mean of 23 days to seek eye care from the onset of symptoms, which may have led to worse visual outcomes.

This study updates knowledge regarding the primary etiological agents of microbial keratitis at a tertiary referral hospital in Sao Paulo and the possible risk factors. A comparison with the Cariello et al.^(10)^ study, which described the epidemiological features of microbial keratitis cases at Hospital Sao Paulo between 1975 and 2007, shows that ocular trauma and contact lens use remain risk factors for fungal and *Acanthamoeba* keratitis, respectively. However, unlike Cariello et al., we found that *C. parapsilosis* was more common among the isolated yeasts than *C. albicans*. To the best of our knowledge, ours is the first study to review the primary etiological agents of infectious keratitis across Brazil. More studies could be done to determine the factors that influence regional differences.
